# Genome-Wide Analysis of Glycoside Hydrolase Family 35 Genes and Their Potential Roles in Cell Wall Development in *Medicago truncatula*

**DOI:** 10.3390/plants10081639

**Published:** 2021-08-10

**Authors:** Junfeng Yang, Qian Li, Wenxuan Du, Yu Yao, Guoan Shen, Wenbo Jiang, Yongzhen Pang

**Affiliations:** 1 Institute of Animal Science, Chinese Academy of Agricultural Sciences, Beijing 100193, China; jfyang63@ibcas.ac.cn (J.Y.); lq798711247@163.com (Q.L.); n053727@163.com (W.D.); jiangwenbo@caas.cn (W.J.); 2 Key Laboratory of Plant Resources and Beijing Botanical Garden, Institute of Botany, Chinese Academy of Sciences, Beijing 100093, China; 3 Chinese Academy of Sciences, Beijing 100049, China; 4 The Institute of Medicinal Plant Development, Beijing, 100193, China; yy474032993@outlook.com (Y.Y.); gashen@implad.ac.cn (G.S.)

**Keywords:** *Medicago truncatula*, *BGAL* genes, cell wall remodeling, expression profile, phylogenetic analysis

## Abstract

Plant β-galactosidases (BGAL) function in various cell wall biogeneses and modifications, and they belong to the glycoside hydrolase family. However, the roles of BGAL family members in *Medicago truncatula* cell wall remodeling remain unclear. In this study, a total of 25 *MtBGAL* members of the glycoside hydrolase gene family 35 were identified, and they were clustered into nine sub-families. Many cis-acting elements possibly related to MeJA and abscisic acid responses were identified in the promoter region of the *MtBGAL* genes. Transcript analyses showed that these *MtBGAL* genes exhibited distinct expression patterns in various tissues and developing stem internodes. Furthermore, a stem-specific expression module associated with cell wall metabolic pathways was identified by weighted correlation network analysis (WGCNA). In particular, *MtBGAL1* and *MtBGAL23* within the stem-specific expression module were highly expressed in mature stems. In addition, several genes involved in lignin, cellulose, hemicellulose and pectin pathways were co-expressed with *MtBGAL1* and *MtBGAL23*. It was also found that *MtBGAL1* and *MtBGAL23* were localized to the cell wall at the subcellular level, indicating their roles in the modification of cell wall metabolites in *Medicago*. As a whole, these results will be useful for further functional characterization and utilization of *BGAL* genes in cell wall modifications aiming to improve the quality of legume forage crops.

## 1. Introduction

β-Galactosidases (BGAL, EC 3.2.1.23) are glucoside hydrolases (GHs) universally distributed in nature and are presented in bacteria, fungi, plants and animals. Based on amino acid sequence similarity, BGALs were grouped into five GH families (GH1, GH2, GH35, GH42 and GH59) [1]. Notably, all BGALs of plant origin were found only in the GH35 family. Typically, they follow the classical Koshland retaining mechanism, releasing galactose molecules via the hydrolysis of terminal β-galactosyl residues, which leads to net retention of the β-anomeric configuration [2]. This process usually requires the participation of two catalytic glutamate residues: one residue acts as a proton donor and the other as a nucleophile [3].

In plants, BGALs have been found to be associated with diverse physiological functions by remodeling (expansion and degradation) the cell wall, including softening of fruit [4,5], development of floral organs [6,7], formation of cellular secondary walls [8], secretion of seed mucilage [9] and elongation of hypocotyl [7]. Furthermore, when the cell wall metabolites that BGALs utilized were analyzed, a wide range of β-D-linked galactosyl residue linkages of cell wall substrates were found, including arabinogalactan proteins [10], xyloglucans [6], galactan [5] and rhamnogalacturonan I [11]. BGALs possess substrate specificity: some BGALs prefer pectic *β*- (1→4) galactan as the substrate, others specifically act on the *β*- (1→3)/(1→6) galactosyl linkages of arabinogalactan proteins [12].

In legumes, alfalfa (*Medicago sativa*) is regarded as the most important and widely cultivated forage species in the world, due to its high biomass yield, exceptional nutritional value and strong resistance [13,14,15]. The cell wall is critical in controlling the growth and morphological uprightness of alfalfa, and modulations of the composition of the secondary cell wall, for example, a reduction in lignin content, can improve the quality of alfalfa with better digestibility and higher fermentable sugar yields for biofuel production [16,17]; interaction hemicelluloses and pectins with cellulose fibrils contribute to the structural integrity and strength of the cell wall [8,18]. Furthermore, the number of side chains of branching galactose residues could impact the binding capacity between these polysaccharides, and could be affected by BGAL enzymes activity [4,7,18]. Therefore, in-depth exploration into the function of BGALs in cell wall processes is essential in alfalfa for quality improvement.

*Medicago truncatula* is diploid with a small genome, short growing period, self-pollination and high genetic transformation efficiency, as well as being a close relative of alfalfa that is a cross-pollinated autotetraploid species [19]. Thus, *M. truncatula* was utilized as a model legume species for genetic analyses [20]. Progresses in *M. truncatula* will certainly contribute to the elucidation of the molecular mechanisms in alfalfa. To date, the BGAL family members in *M. truncatula* have not been reported and their biological functions remain unclear. In the current study, a search for *BGAL* genes in the *M. truncatula* genome identified 25 BGAL gene members. We further performed a set of analyses, including sequence analysis (phylogenetic tree, gene structure and motifs composition, gene distribution, synteny relationship and cis-acting elements), analyses on specific tissues and dynamic expression patterns of *MtBGALs* during stem maturation, and gene location. This study provides a valuable reference to further elucidate the role of *BGAL* genes in cell wall remodeling in legume plants.

## 2. Results

### 2.1. Identification and Analyses of BGAL Gene Family Members in M. truncatula

A combination of homology search and PFAM analysis resulted in the identification of 25 candidate *M. truncatula BGAL* genes, and they were designated as *MtBGAL1* to *MtBGAL25* based on their location on the chromosome (Table 1). The majority of the deduced MtBGAL proteins (19 of 25) contained predicted signal peptides, generally the first 19–29 amino acids. The predicted mature MtBGAL proteins contained amino acids of 228 (MtBGAL22) to 907 (MtBGAL25), with corresponding molecular weights of 26.11 to 101.17 kDa. Except MtBGAL22, all the other MtBGAL proteins contain 1 to 16 N-glycosylation sites. Isoelectric points of the predicted proteins ranged from 5.15 to 9.42. The majority of the deduced MtBGAL proteins were predicted to be localized in the cell wall. However, only MtBGAL22 and MtBGAL24 that lack the N-terminal signal peptide were predicted to be respectively localized in the chloroplast/nucleus or cell membrane (Table 1).

### 2.2. Multiple Sequence Alignment, Phylogenetic Analysis and Classification of the MtBGAL Genes

To understand the evolutionary relationship of BGAL proteins among *Medicago* and other plants (*Arabidopsis thaliana, Oryza sativa, Solanum lycopersicum* and *Prunus persica*), a multiple sequence alignment analysis was performed (Figure S1). All MtBGALs contain the two GH35 domains with two active site glutamate residues: one acts as a proton donor (Q-G-G-P-I-I-L-S/A-Q-I-E-N-E-Y), and the other as a nucleophile (P-N-S/K-P-N/V-K-P-K-M-W-T-E-N-W), except MtBGAL17/19 (lacks proton donor) and MtBGAL7/17/19/22/24 (lacks nucleophile). Apart from the GH35 domain, most MtBGALs also carry an additional Gal_lectin domain in the C-terminus and a so-called GHD domain (beta sandwich-like domain in beta-galactosidase) (Figure 2a). 

**Figure 1:**
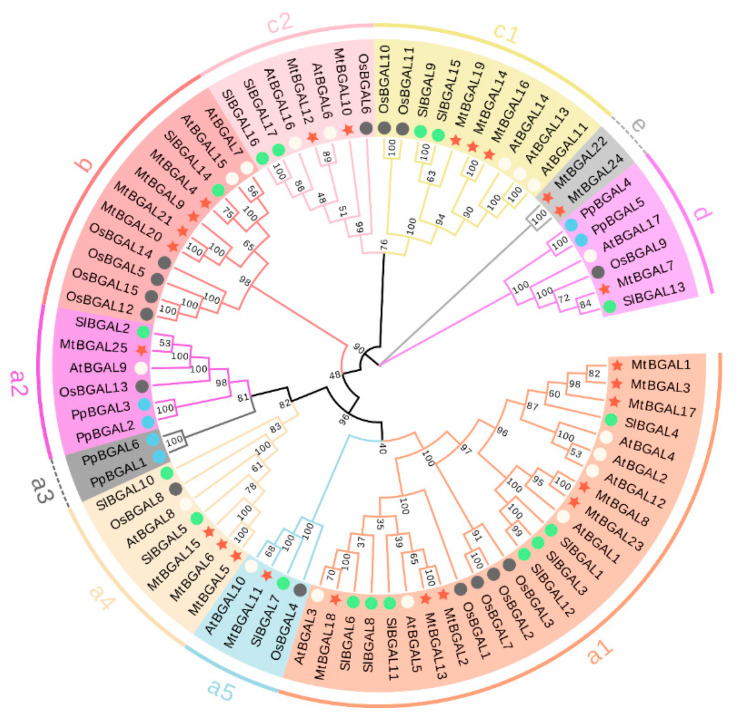
Phylogenetic tree of BGAL proteins from *Medicago, Arabidopsis*, rice, tomato and *Physcomitrella patens*. The neighbor-joining (NJ) method of the MEGA-X program was used to construct the phylogenetic tree; bootstrap was 1000 replicates. The different-colored arcs indicate different groups of BGAL proteins. The red stars, white circles, gray circles, green circles and blue circles represent BGAL proteins from *Medicago, Arabidopsis*, rice, tomato and *P. patens*, respectively.

An unrooted phylogenetic tree was constructed with BGALs from *A. thaliana*, *O. sativa*, *S. lycopersicum* and *P. persica*, and it was found that MtBGALs were presented in all of the BGAL sub-families, except for a bryophyte-specific cluster (a3). In particular, another cluster (e) is species-specific for *Medicago* with only *MtBGAL22* and *MtBGAL24* (Figure 1). In addition, all other clusters of a1, a2, a4, a5, b, c1, c2 and d contain members from *Medicago*, *Arabidopsis*, rice and tomato. 

### 2.3. Analyses of Gene Structure, Conserved Domain and Motif Pattern of MtBGAL Genes

**Figure 2:**
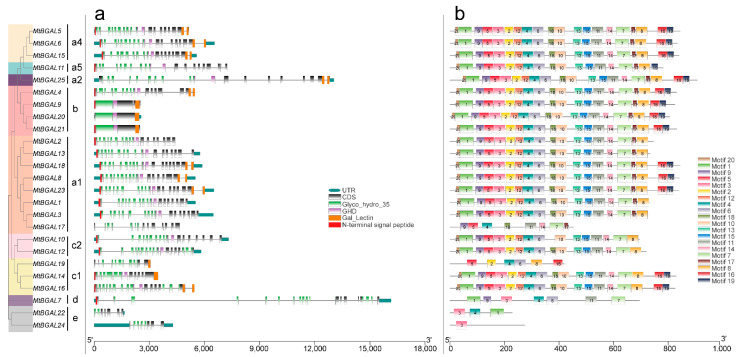
Gene structure and architecture of the conserved protein motifs of 25 *MtBGAL* genes. (**a**) Sequence structure distribution of *MtBGAL* genes; blue boxes indicate 5′- and 3′-untranslated regions, while gray boxes and gray lines indicate exons and introns, respectively. The BGAL domain (glyco_hydro_35), GHD, Gal_lectin and N-terminal signal peptide are highlighted with green, purple, orange and red boxes, respectively. Exon–intron structure and conserved domain of *MtBGAL* genes. Blue boxes indicate 5′- and 3′-untranslated regions; yellow boxes indicate exons; and black lines indicate introns. The BGAL domain (glyco_hydro_1) and N-terminal signal peptide are highlighted by green and red boxes, respectively. (**b**) Conserved BGAL protein motif composition in *Medicago* predicted by MEME. Colored boxes indicate different motifs.

The exon/intron structures of all the identified *MtBGAL* genes of the GH35 family members were examined to gain more insight into the evolution of the *BGAL* genes in *Medicago*. Among all the *MtBGAL* genes, 17 genes (68%) contain 17 to 19 exons, and five (20%) contain 7 to 14 exons. Notably, three members in class b (*MtBGAL9*, *MtBGAL20* and *MtBGAL21*) only have one or two exons (Figure 2a). For the analysis of the GH35 domain, the shortest conserved amino acid length is 59 aa for *MtBGAL22*, and the longest is 327 aa for *MtBGAL7* (Figure 2a).

We used MEME motif analysis to search the conserved motifs in the 25 *Medicago BGAL* genes. As shown in Figure 2b, a total of 20 conserved protein motifs were obtained, which ranged from 11 to 50 amino acids. Motif 3 and motif 12 corresponded to the conserved GH35 domain with two active site glutamate residues, together with motifs 1, 2, 4, 5, 6, 9, 18 and 20 to form the complete GH35 domain. In addition, motif 16 was annotated as Gal_lectin domain, and motif 10 as GHD domain. It showed that over half of the MtBGAL proteins (13 out of 25) contained all these 20 motifs (Figure 2b), but the remaining MtBGAL proteins lacked one or more of these motifs: MtBGAL11 lacked motif 16, MtBGAL1/2/3/10/12/13 lacked motif 16 and 19 (Figure 2b). All these analyses on gene structure, conserved domain and motif pattern demonstrated that the deduced BGAL proteins of the GH35 family in *M. truncatula* are relatively conservative.

### 2.4. Chromosomal Distribution and Synteny Analysis of the MtBGAL Genes

**Figure 3:**
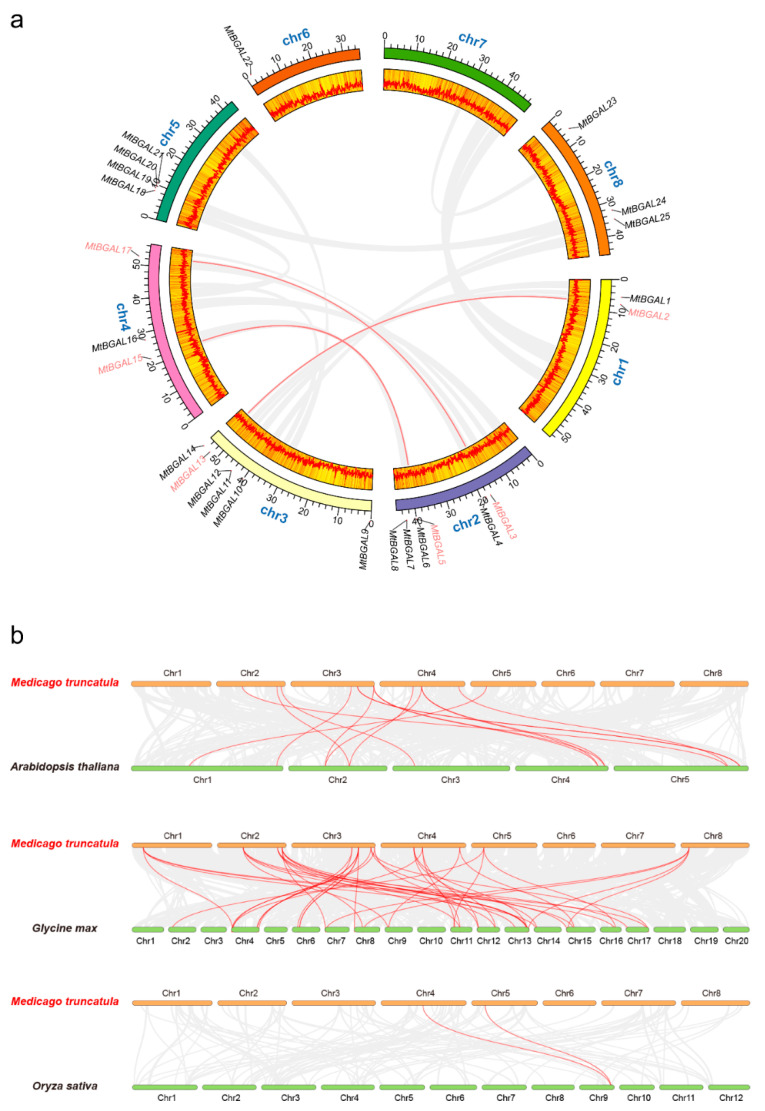
Chromosome distribution and syntenic analysis of BGAL genes in *M. truncatula*. (**a**) The chromosomal distribution and interchromosomal relationships of *MtBGAL* genes. The segmentally duplicated genes are marked in red and connected by red curves. (**b**) Synteny analysis of *BGAL* genes between *Medicago* and three representative plant species (*A. thaliana, G. max and O. sativa*). Collinear blocks in the background within *Medicago* and *A. thaliana/G. max/O. sativa* are shown with gray lines, and the syntenic *MtBGAL* gene pairs are shown with red lines.

The 25 *MtBGAL* genes were randomly distributed on seven chromosomes in *Medicago* except chromosome 7 (Figure 3a). More *MtBGAL* genes localized on chromosomes 2 and 3 than on others, with only one gene on chromosome 6. During the evolution, both tandem and segmental duplication contribute to the generation of gene family [21]. However, no tandem repeat event was found for *BGAL* genes in the *Medicago* genome (Figure 3a). Only three segmental duplication events were identified for six *MtBGAL* genes: MtBGAL2/13, MtBGAL3/17 and MtBGAL5/15.

To further explore the phylogenetic mechanism of the *MtBGAL* gene family, comparative syntenic maps between *Medicago* and the three representative species *Arabidopsis*, soybean and rice were constructed (Figure 3b). A total of 18 *MtBGAL* genes showed syntenic relationship with those in *Arabidopsis* (11), soybean (15) and rice (2) (Additional file S1). Fifteen, fifty-two and two orthologous pairs were identified between *Medicago* and *Arabidopsis*, *Medicago* and soybean and *Medicago* and rice, respectively. Some *MtBGAL* genes were found to be associated with six syntenic gene pairs between *Medicago* and soybean, such as *MtBGAL2* and *MtBGAL13*, and these genes might play an important role during evolution. Interestingly, one collinear gene pair (*MtBGAL19/OsBGAL11*) identified between *Medicago* and rice was not found between *Medicago* and *Arabidopsis*, or *Medicago* and soybean, implying that the orthologous pair formed after the divergence of dicotyledonous and monocotyledonous plants. Besides, the collinear pair of *MtBGAL16* was identified between *Medicago* and *Arabidopsis*, *Medicago* and soybean, and *Medicago* and rice, indicating that these collinear pairs may have already existed before the ancestral divergence.

To better comprehend the evolutionary stress acting on the formation of the BGAL gene family, the Ka/Ks value of the *BGAL* gene pairs were calculated (Additional file S1). All segmental duplicated *MtBGAL* gene pairs, and the majority of the orthologous MtBGAL gene pairs (except *MtBGAL14/AtBGAL14, MtBGAL16/AtBGAL14* and *MtBGAL16/OsBGAL11*), had a Ka/Ks value of less than 0.35, indicating that *MtBGAL* genes might have experienced strong purifying selective pressure during evolution. Meanwhile, the low Ka/Ks value also suggests that the gene probably keeps its function, however, putative increase does not necessarily mean that the gene is degenerating.

### 2.5. Analysis of the Cis-Acting Elements in the Promoter Regions of MtBGAL Genes

**Figure 4:**
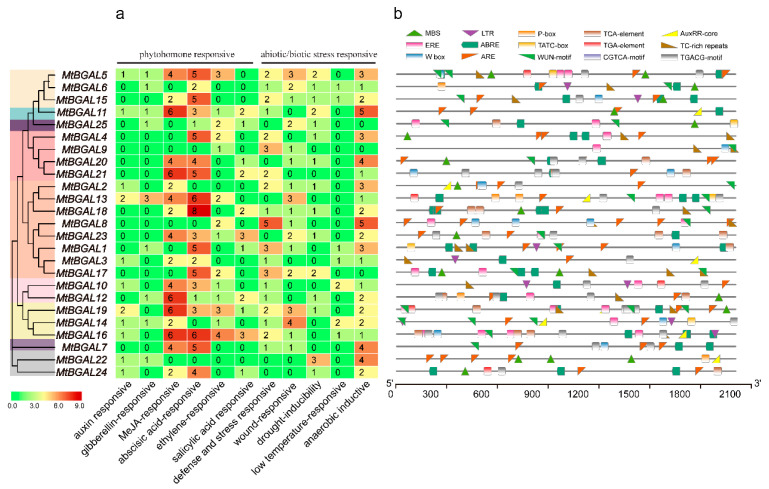
Prediction of cis-acting elements in the promoter region of *MtBGALs*. (**a**) The numbers of cis-acting elements detected in the promoter region of each *MtBGAL* gene, which is presented in the form of a heat map. (**b**) Colored polygon represented different types of cis-acting elements and their relative location for each *MtBGAL* gene.

The cis-acting elements in the upstream promoter regions of the *MtBGAL* genes were analyzed. Among them, six hormone-responsive cis-acting elements (auxin, gibberellin, MeJA, abscisic acid, ethylene and salicylic acid), and five abiotic/biotic stress-responsive cis-acting elements (stress, wound, drought, low-temperature and anaerobic) were predicted (Figure 4, Additional file S2). Except for *MtBGAL3/6/9/22/25*, all the other *MtBGALs* possessed at least 10 stress- and hormone-responsive related cis-acting elements. In particular, the most abundant *cis*-acting elements for all the *MtBGAL* genes were MeJA- (TGACG-motif, CGTCA-motif) and abscisic-acid- (ABRE-motif) responsive elements. These results indicated that *MtBGAL* genes might be involved in various stress responses and hormone pathways by participating in different processes.

### 2.6. Analysis of Co-Expression Network of Cell Wall Genes with WGCNA

**Figure 5:**
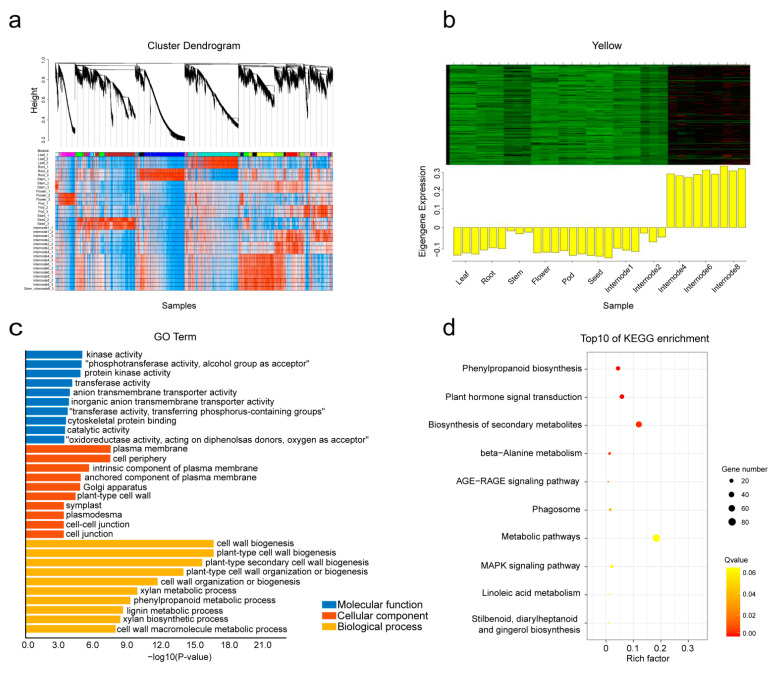
WGCNA of co-expression genes. (**a**) Hierarchical cluster tree showing co-expression modules identified by WGCNA. Each leaf corresponds to an individual gene. The branches correspond to modules of highly interconnected genes. The color rows below the dendrograms indicate different module memberships. (**b**) Heatmaps showing the expression profile of all the co-expressed genes in the stem-specific module. Each row in the heatmap corresponds to an individual gene. Bar graphs (below the heat maps) indicate the consensus expression pattern of the co-expressed genes within each module. (**c**) Analysis of enriched GO terms for the gene sets within the stem-specific module. (**d**) KEGG enrichment analysis of gene sets of the stem-specific module. The larger the circle is, the more genes are enriched in the metabolic pathway, and the more intensive the color is, the more significant the metabolic pathway is.

BGALs are known to play important roles in plant growth and development through cell wall remodeling [4,6,22]. To investigate the gene regulatory network during cell wall development, a total of 25,604 differentially expressed genes (with available GeneChip probe sets) of six tissues (root, stem, leaf, flower, pod and seed) and five developing stem internodes (internodes 2, 3, 5, 7, and 9 from the primary stem) were collected to construct the co-expression network with WGCNA. A total of 20 distinct modules (labeled in different colors) were identified (Figure 5a). The 20 distinct modules represented the collection of genes with distinct tissue-specific expression patterns. In particular, a stem-specific module was identified (containing 1964 genes), which showed the highest expression level in stems, especially in mature stems (Figure 5b). 

To identify the biological processes in which the stem-specific module was involved, the Gene Ontology (GO) and KEGG pathway enrichment were analyzed at a significance level of *p* < 0.05. GO analysis revealed enriched metabolic processes including cell wall biogenesis, secondary cell wall biogenesis, cell wall organization or biogenesis (Figure 5c). The KEGG annotations indicated that phenylpropanoid biosynthesis, plant hormone signal transduction and biosynthesis of secondary metabolites were enriched (Figure 5d). Thus, GO and KEGG enrichment analysis together indicated that the cell wall metabolic pathway was closely related to the stem-specific module.

### 2.7. Expression Profiles of the MtBGAL Genes and Identification of Stem-Specific MtBGAL Genes

**Figure 6:**
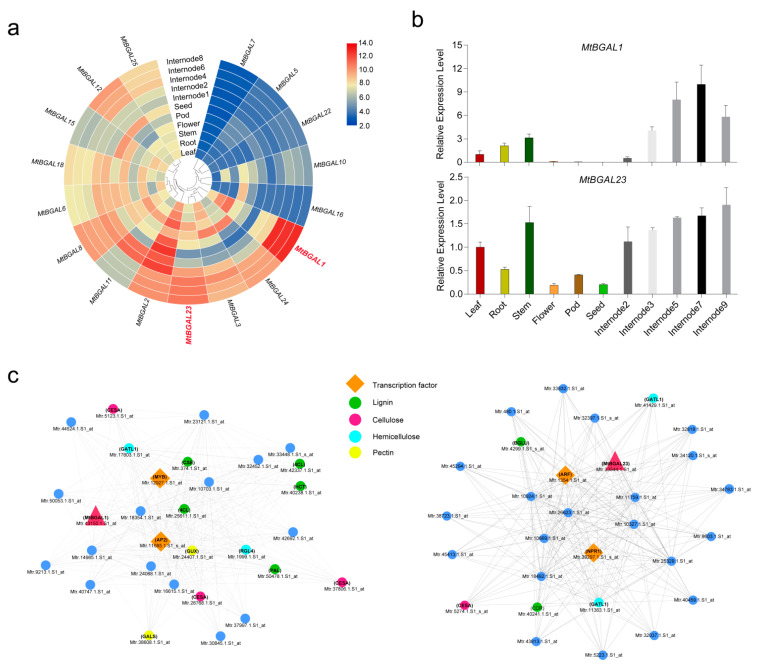
Expression profiles of *MtBGAL* genes. (**a**) Expression profiles of *MtBGAL* genes in six different tissues and five developing internodes from microarray data. (**b**) Expression analysis of *MtBGAL1* and *MtBGAL23* in six representative samples and different stem internodes by qPCR analysis. (**c**) The correlation network of *MtBGAL1* and *MtBGAL23* in the stem-specific module by using Cytoscape. *MtBGAL1* and *MtBGAL23* were shown in red triangle, transcription factors were shown in orange rhombus, and enzyme genes in lignin, cellulose, hemicellulose and pectin pathway were presented in blue, red, cyan and yellow, respectively.

To investigate the expression patterns of the *MtBGAL* gene family in various tissues, a total of 58 probes corresponding to 17 *MtBGAL* genes (68%) (with available probe sets from GeneChip data) were identified; one representative probe for each gene was selected for expression analysis (Additional file S3). Generally, distinct tissue-specific expression profiles were found for *MtBGAL* genes (Figure 6a). Notably, *MtBGAL1* and *MtBGAL23* specifically expressed in stems, and their highest expression levels were found in mature stems. In particular, these two *MtBGAL* genes were also presented within the stem-specific module (Figure 6a). To verify the expression profiles of *MtBGAL1* and *MtBGAL23* from GeneChip data, qPCR analyses were performed with different tissues and different stem internodes. It was clear that *MtBGAL1* and *MtBGAL23* showed the highest transcript level in stems, and the expression levels in the 5th to 9th stem internodes were higher than that in the 2nd to 3rd internodes (Figure 6b), which was consistent with the results of the GeneChip data. These data strongly suggest that *MtBGAL1* could be involved in secondary cell wall synthesis.

In order to further investigate the biological function of *MtBGAL1* and *MtBGAL23*, two co-expression networks were constructed by using *MtBGAL1* and *MtBGAL23* as bait genes. The genes of this network were screened from the stem-specific module with a Pearson correlation coefficient of larger than 0.95 (Figure 6c). It was found that a large number of genes involved in lignin (*PAL*, *4CL*, *CSE*, *HCT*, *BGLU* and *CCR*), cellulose (*CESA*), hemicellulose (*GATL1* and *RGL4*) and pectin (*GUX* and *GALS*) biosynthesis were presented in the co-expression network with *MtBGAL1* (Figure 6c, left) and *MtBGAL23* (Figure 6c, right). In addition, some related transcription factor genes (*MYB*, *AP2*, *ARF* and *NPR1*) were also found in these networks, indicating that they may regulate the metabolic pathway that *MtBGAL1* and/or *MtBGAL23* are involved in. These results suggested that *MtBGAL1* and *MtBGAL23* are possibly involved in cell wall metabolism.

### 2.8. Subcellular Localization of MtBGAL1 and MtBGAL23

**Figure 7:**
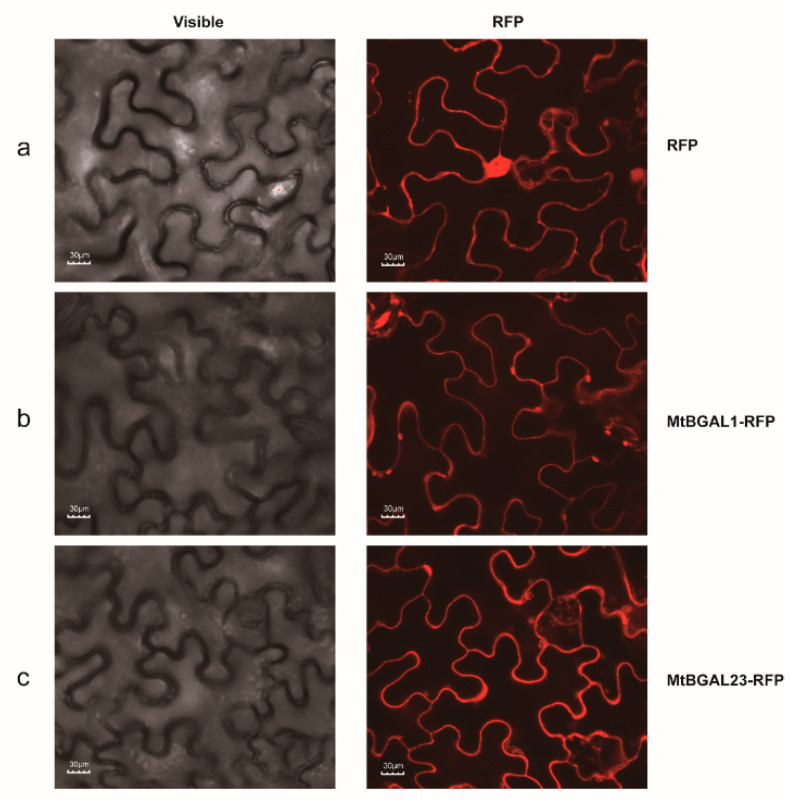
Subcellular localization of *MtBGAL1* and *MtBGAL23*. Confocal microscopy images of *N. benthamiana* epidermal leaf cells transiently expressing MtBGAL1-RFP/MtBGAL123-RFP fusion protein (**b**,**c**), together with the vector control (**a**); (**a**–**c**): left panels, blight field; middle panels, merged with red fluorescence; and scale bars = 30 μm.

In order to further explore the characteristics of *MtBGAL1* and *MtBGAL23*, we analyzed their subcellular localization by transient expression in *N. benthamiana* epidermal cells in comparison with the vector control. The cells transformed with vector control with RFP displayed clear red fluorescence signals in the whole cell, in particular in the cytosol (Figure 7a). The cells’ transformed constructs containing MtBGAL1-RFP and MtBGAL23-RFP fusion protein displayed strong red fluorescence signals in the cell wall of the *N. benthamiana* epidermal cell (Figure 7b,c). Together, these results indicated that both MtBGAL1 and MtBGAL23 are predominantly localized in the cell wall, which is consistent with their tissue-specific expression pattern in the stem.

## 3. Discussion

β-galactosidases are widely distributed in plants and are associated with a diverse assortment of biological processes, including plant growth, fruit softening, pollen development and seed germination [4,6,11,23]. Here, we identified 25 putative *BGAL* genes from *Medicago*, which were characterized in sequence features, phylogenetic relationships, gene structures, chromosomal localization, synteny analysis and gene expression profiles.

The majority of the deduced MtBGAL proteins, except MtBGAL7/17/19/22/24, contained the two putative active-site-containing consensus sequences: QGGPIILS/AQIENEY and PNS/KPN/VKPKMWTENW [3], and the length of the polypeptides varied from 695 to 907 aa (Table 1). The length of BGAL from *M. truncatula* is consistent with *A. thaliana* and *O. sativa* that vary from 697 to 988 aa and 673 to 956 aa, respectively [24,25]. Therefore, these 20 MtBGALs (except MtBGAL7/17/19/22/24) with active site consensus Glu residues and typical sequence length seemed to be able to produce active β-galactosidases. Furthermore, all characterized BGALs in *Arabidopsis* to date are localized in the cell wall [22]. Similarly, in *M. truncatula*, most MtBGAL members showed predicted localization to the cell wall (except *MtBGAL22* and *MtBGA24*), implicating their potential role in cell wall remodeling and expansion through catalyzing the substrates that are sequestrated on the cell wall (e.g., galactans arabinogalactans, arabinogalactan proteins and xyloglucans) [26].

Combining gene expression patterns and phylogenetic and synteny analysis could throw light on the functional roles of *MtBGAL* genes that are involved in specific physiological processes. For example, *MtBGAL12* was highly expressed in seeds rather than in other tissues, and its ortholog gene of *Arabidopsis*, *AtBGAL6*, is also expressed predominantly in seed coats where it alters the hydration properties of mucilage by modifying carbohydrate structures [9]. The similar expression pattern indicated that *MtBGAL12* may also be required for the production of seed coat mucilage during seed germination in *Medicago*. *MtBGAL11* is the sole representative in *Medicago* of subfamily a5, and its ortholog in *Arabidopsis*, *AtBGAL10*, was also the only member of subfamily a5 in *Arabidopsis*. Meanwhile, *AtBGAL10* acts on xyloglucan cell wall substrates, and the *Atbgal10* mutant showed shorter siliques and sepals than the wild type [6]. Accordingly, we inferred that the ortholog gene of *AtBGAL10*, *MtBGAL11,* may also participate in cell wall modification in flowers of *M. truncatula*. Another two *MtBGALs* (*MtBGAL8* and *MtBGAL18*) in cluster a1 were highly expressed in stems, and they were close to *AtBGAL1* and *AtBGAL3* in the phylogenetic tree. *AtBGAL1* and *AtBGAL3* act in coordinated ways during cell elongation by increasing galactose levels by analyzing the *bgal1/bgal3* double mutant of *Arabidopsis* [7], thus their ortholog genes *MtBGLU8* and *MtBGLU18* may share similar function in the maintenance of the cell wall architecture in *Medicago*. In addition, silencing of *SlBGAL4* (subfamily a1) in tomato resulted in decreased fruit softening [4], therefore the *MtBGALs* in subfamily a1 may have similar roles in pod development in *Medicago*.

It was reported that several *BGAL* genes were also associated with various physiological processes during cell wall remodeling [8,12,25]. In *A*. *thaliana*, five BGAL proteins of the subfamily a1 were localized in the cell wall, and they were associated with many developmental processes during cell wall extension, stiffening or vegetative organ elongation [7,27,28]. Similarly, 4 *BGALs* from chickpeas were required for galactan remodeling during cell division and elongation, secondary cell wall deposition or fiber differentiation [29,30,31]. In flax, *BGAL* was demonstrated to be necessary for the dynamic remodeling of polysaccharides that occurs during normal secondary cell wall development in fibers [8]. Notably, the composition of the secondary cell wall significantly affects the quality of legume forage [17,32].

Therefore, in this study, the expression profiles of secondary-cell-wall-related genes during stem maturation were analyzed by WGCNA in *M. truncatula*, and a stem-specific module which correlated with secondary cell wall development was identified (Figure 5). Some genes in this module have previously been demonstrated to be involved in secondary cell wall metabolism, including *MtNST1* (NAC secondary cell wall thickening promoting) [33], *MtCCR1* (cinnamoyl CoA reductases) [34] and *MtIRX1* [34]. In another systematic study on the expression analyses of secondary cell wall development in *Medicago truncatula,* genes encoding b-ZIP, NAC, WRKY, C2H2 zinc finger (ZF), homeobox and HSF transcription factors were also presented and predicted to be putative regulators of secondary cell wall development [35]. In our present study, *MtBGAL1* and *MtBGAL23*, two members of the sub-family a1, were also found in this module and co-expressed with a variety of lignin, cellulose, hemicellulose and pectin biosynthesis-related genes (Figure 6). More importantly, transient expression in *N. benthamiana* showed that *MtBGAL1* and *MtBGAL23* are localized in the cell wall (Figure 7). All these pieces of evidence demonstrated that *MtBGAL1* and *MtBGAL23* are involved in the metabolism of secondary cell wall in *M. truncatula*, and this screen strategy seems to be effective for new gene discovery, although the detailed function requires further elucidation.

Analysis on diverse cis-acting elements could provide important information on functional characterization of *MtBGAL* genes. In this study, most members in the GH35 family contained several MeJA-responsive, ABA-responsive, and anaerobic-inductive *cis*-elements, indicating that these *MtBGAL* genes may play roles in cell wall remodeling through association with MeJA, ABA or anaerobic induction. In apples, the promoter activities and transcript level of *Mdβ*-*Gal2* were induced by both MeJA and ethylene treatment, which were involved in different fruit textures of apple cultivars by influencing the degradation of pectin during fruit maturity [36]. In *M. truncatula*, four β-glucosidase genes were also reported to be involved in the turnover of formononetin glucoside, which were induced by MeJA signals in medicarpin synthesis [37]. Multiple MeJA-responsive cis-acting elements were presented in *MtBGAL* genes, with four for the *MtBGAL23* gene, highlighting that *MtBGAL23* is likely induced by MeJA during cell wall development. In general, this study provides insight into the potential roles of *MtBGAL* genes involved in cell wall remodeling, yet further explorations on the biochemical and genetic function of *MtBGALs* are required.

## 4. Materials and Methods

### 4.1. Identification of MtBGAL Genes of the GH35 Family in the M. truncatula Genome

Hidden Markov model (HMM) profiles of Glyco_hydro_35 (PF01301) were downloaded from the Pfam database (https://pfam.xfam.org/, accessed on 15 March 2020) and used as the query (*p* < 1 × 10^−5^) to search the *BGAL* genes from the *M. truncatula* genome database. Furthermore, the *BGAL* genes in *A. thaliana* were downloaded from the TAIL database (https://www.arabidopsis.org/, accessed on 15 March 2020), which were employed as a query to blast against the *M. truncatula* genome database. All candidate *MtBGAL* genes that may contain BGAL domain based on HMMER and the BlastP results were further confirmed by the presence of the BGAL core sequences using the InterProScan (https://www.ebi.ac.uk/interpro/search/sequence-search, accessed on 15 March 2020), CDD (https://www.ncbi.nlm.nih.gov/Structure/bwrpsb/bwrpsb.cgi, accessed on 15 March 2020) and SMART (http://smart.embl-heidelberg.de/, accessed on 15 March 2020) program. In total, 25 candidate *MtBGAL* genes were obtained and assigned based on their locations on chromosomes (Table 1). 

### 4.2. Sequence Analyses and Structural Characterization of the MtBGAL Genes

All the sequences of *MtBGAL* genes were submitted to the ExPASy website (http://web.expasy.org/protparam/, accessed on 15 March 2020) to calculate the number of amino acids, molecular weight (MW) and theoretical isoelectric points (pI), and they were further analyzed using the ProtParam tools. Their signal sequences and N-glycosylation sites were predicted by using SignalP (http://www.cbs.dtu.dk/services/SignalP, accessed on 15 March 2020) [38] and NetNGlyc 1.0 Server (http://www.cbs.dtu.dk/services/NetNGlyc/, accessed on 15 March 2020), respectively. Cellular locations of MtBGAL proteins were predicted by using Plant-mPLOC (http://www.csbio.sjtu.edu.cn/bioinf/plant-multi/, accessed on 15 March 2020). Sequence alignments and dendrograms in homeodomain sequences of the BGAL proteins were analyzed with Jalview (http://www.jalview.org/Web_Installers/install.htm, accessed on 15 March 2020). Conserved motifs in MtBGAL protein sequences were identified by the MEME program (http://meme-suite.org/, accessed on 15 March 2020) [39] using the default settings, except the motif number was set to 20. The visualization of exon–intron position was executed through Amazing Optional Gene Viewer software [40].

### 4.3. Phylogenetic Analysis and Classification of the MtBGAL Genes

The full-length amino acid sequences of BGALs derived from *Arabidopsis* [24], rice [25], tomato [41], flax [42] and *Physcomitrella patens* [43], together with the MtBGALs, were used for phylogenetic analysis. All of these sequences were firstly aligned by using ClustalX with the default parameters. Subsequently, an unrooted neighbor-joining phylogenetic tree was constructed based on the neighbor-joining method (with 1000 bootstrap replicates) using MEGA-X software [44].

### 4.4. Analyses of the Chromosomal Distribution and Gene Duplication of the MtBGAL Genes

All *MtBGAL* genes were mapped to the eight *M. truncatula* chromosomes based on physical location information from the database of *Medicago* genome using Circos [40]. Multiple Collinearity Scan toolkit (MCScanX) was used to analyze the gene duplication events with default parameters [45]. Their intraspecific synteny relationship in *M. truncatula* was analyzed by Amazing Gene Location software [40]. To exhibit the interspecific synteny relationship between *M. truncatula* and the other two representative model plant species (*Arabidopsis* and rice), the syntenic maps were constructed using the Dual Systeny Plotter software [40]. Non-synonymous (ka) and synonymous (ks) values of *MtBGAL* homologous gene pairs were calculated using Simple Ka/Ks Calculator software [40].

### 4.5. Analysis of Cis-Acting Elements in the Promoter Region of the MtBGAL Genes

The cis-acting elements were predicted from the 2 kb upstream promoter sequences of the *MtBGAL* genes that were uploaded to PlantCARE (http://bioinformatics.psb.ugent.be/webtools/plantcare/html/, accessed on 15 March 2020) [46]. 

### 4.6. Analysis on Gene Co-Expression Network and GO/KEGG Enrichment

Expression data were collected from the *M. truncatula* Gene Expression Atlas (https://Mtgea.noble.org/v3/, accessed on 15 March 2020). Data for root, stem, leaf, flower, pod, seed and different stem internodes (33 GeneChips in total) were used. First, only the probe sets with “Mtr” for *M. truncatula* were left. Then, the probe sets showing very low expression levels (RPKM less than 10) were removed. Finally, probes with different expression levels were identified by comparing different tissues and stem internodes using DEseq by multiple-factor design. After these filtering steps, 25,604 differential expressed genes still remained. The co-expression gene network for those selected probe sets were constructed using WGCNA. The modules were obtained using the automatic network construction function blockwise modules with default settings, except that the power is set at 16, TOMType is employed, minModuleSize is 30 and mergeCutHeight is 0.25.

Gene Ontology enrichment analysis for gene sets of the stem-specific module was performed using TBtools [40]. The GO terms with a corrected (after adjusting with false discovery rate) *p*-value of ≤ 0.05 were considered to be significantly enriched. KEGG enrichment analysis of gene sets of the stem-specific module was performed using KOBAS 3.0 (http://kobas.cbi.pku.edu.cn/kobas3, accessed on 15 March 2020) (significance value ≤ 0.05).

### 4.7. Plasmid Construction and Agrobacterium-Infection Assays in Nicotiana Benthamiana

We cloned the open reading frames of *MtBGAL1* and *MtBGAL23* genes with gene-specific primers as listed in Table S4, and constructed them into the botany expression vector pCAMBIA 1302 fused with red fluorescent protein (RFP). The resulting vector pCAMBIA 1302 was transformed into *Agrobacterium tumefaciens* strain GV3101 for infiltration. The *Agrobacterium* were cultured and re-suspended in the inoculation buffer (10 mM MgCl_2_, 2 mM acetosyringone, 100 mM MES (pH 5.7)) for 10 min at room temperature. The suspensions were then adjusted to OD_600_ = 0.8 and were infiltrated into leaves of 4-week-old *N. benthamiana* plants with needleless syringes.

### 4.8. Plant Materials

*M. truncatula* (cv. Jemalong A17) plants were used in this study. The stems, roots, leaves, flowers, pods (20-day-old pods) and seeds (20-day-old seeds) of mature *M. truncatula* plants were collected separately for RNA extraction and for further qPCR analysis. To investigate the expression pattern of MtBGAL1 in different stem internodes, plants were grown in a growth chamber at 24 °C with 16 h light/8 h dark photoperiod. Stems of 7-week-old *M. truncatula* plants (a total of 10–11 internodes) were used for sampling as previously reported [35]. Internodes 2, 3, 5, 7 and 9 counting from the top were collected, and immediately frozen in liquid nitrogen and stored at −80°C for subsequent analysis.

### 4.9. RNA Extraction and Gene Expression Analysis

Total RNAs were isolated using Eastep Super total RNA Extraction kit (Promega, Shanghai, China) according to the manufacturer’s instructions; first-strand cDNA synthesis was performed using Trans Script One-Step gDNA Removal and cDNA Synthesis SuperMix (TransGen Biotech, Beijing, China) according to the manufacturer’s instructions. Quantitative real-time PCR (qRT-PCR) was performed using a 2 RealStar Green Fast Mixture (GeneStar, Shanghai, China) and ABI 7500 real-time Detection System (Applied Biosystems, Foster City, CA, USA) according to our previous report [47]. The procedure used for qRT-PCR was 30 s at 94 °C, followed by 40 cycles of 5 s at 94 °C and 34 s at 60 °C. The housekeeping gene of actin-related protein 4A gene was used as a reference gene for the relative expression patterns analysis. The reactions were performed with three biological replicates and the data were analyzed using the 2^−^^ΔΔCT^ method. The results were analyzed by means ± standard deviation (SD). The primers for qRT-PCR are listed in Table S4.

## 5. Conclusions

Twenty-five *MtBGAL* genes of the GH35 family were identified and characterized in *M. truncatula* in the present study, and they were further classified into nine clusters. Phylogenetic relationship and synteny analysis on *MtBGAL* genes from several different plant species provided valuable information on the evolutionary characteristics of *MtBGAL* genes. Moreover, analyses of their expression profiles in different tissues and internodes based on GeneChip data and qPCR validation, combined with subcellular localization, indicated that *MtBGAL1* and *MtBGAL23* may contribute to secondary cell wall remodeling during stem maturation in *M. truncatula*. Our results provide prerequisite information for further functional characterization of individual *BGAL* genes in *M. truncatula*.

## Figures and Tables

**Table 1:** Properties and locations of the predicted BGAL proteins in *M. truncatula*.

Gene Name	Gene ID	Pre-Protein			Mature Protein				
		Aa	MW	Cleavage Site	Aa	MW	Theoretical pI	N-gly Site	Possible Destination
MtBGAL1	Medtr1g018200	731	81.73	29–30	702	78.43	5.59	3	Cell Wall
MtBGAL2	Medtr1g023120	746	83.19	27–28	719	80.21	8.60	1	Cell Wall
MtBGAL3	Medtr2g039120	727	81.57	26–27	701	78.43	8.74	2	Cell Wall
MtBGAL4	Medtr2g042610	831	93.31	24–25	807	90.58	5.53	10	Cell Wall
MtBGAL5	Medtr2g094020	844	93.21	19–20	825	90.98	6.73	4	Cell Wall
MtBGAL6	Medtr2g094060	834	91.36	19–20	815	89.12	7.48	4	Cell Wall
MtBGAL7	Medtr2g100000	695	78.76	29–30	666	75.63	7.16	4	Cell Wall
MtBGAL8	Medtr2g100110	842	93.16	26–27	816	90.44	6.52	1	Cell Wall
MtBGAL9	Medtr3g005570	825	91.99	23–24	802	89.65	6.48	6	Cell Wall
MtBGAL10	Medtr3g088520	695	77.58	23–24	672	74.97	8.97	4	Cell Wall
MtBGAL11	Medtr3g096900	782	87.55	21–22	761	85.28	7.86	5	Cell Wall
MtBGAL12	Medtr3g096910	721	80.87	23–24	697	78.31	5.15	12	Cell Wall
MtBGAL13	Medtr3g112370	734	81.31	26–27	708	78.62	8.66	3	Cell Wall
MtBGAL14	Medtr3g117840	829	92.25	23–24	806	89.92	8.65	3	Cell Wall
MtBGAL15	Medtr4g059680	840	91.38	23–24	817	88.86	6.21	4	Cell Wall
MtBGAL16	Medtr4g073290	826	92.48	21–22	805	90.26	8.31	3	Cell Wall
MtBGAL17	Medtr4g126330	453	51.02	-	-	-	9.42	3	Cell Wall
MtBGAL18	Medtr5g021190	844	93.90	24–25	820	91.27	6.88	1	Cell Wall
MtBGAL19	Medtr5g022590	419	48.09	-	-	-	8.70	1	Cell Wall
MtBGAL20	Medtr5g024080	807	90.33	-	-	-	6.78	16	Cell Wall
MtBGAL21	Medtr5g025830	832	93.12	25–26	807	90.40	8.28	6	Cell Wall
MtBGAL22	Medtr6g007470	228	26.11	-	-	-	7.62	0	Chloroplast, Nucleus
MtBGAL23	Medtr8g016230	839	92.65	23–24	816	90.25	8.67	1	Cell Wall
MtBGAL24	Medtr8g076800	274	30.89	-	-	-	8.69	1	Cell Membrane
MtBGAL25	Medtr8g085210	907	101.17	-	-	-	7.02	6	Cell Wall

## Data Availability

All data in the present study are available in the public database as referred in the Material and Method part.

## Supplementary Materials

The following are available online at https://www.mdpi.com/article/10.3390/plants10081639/s1, Figure S1: Alignment of multiple MtBGAL amino acid sequences. The sequence conservation is shown as a percentage bar—score below. At the bottom, the sequence logo is shown, summarizing the occurrence of given amino acids at specific positions. Two well-conserved active glutamate residues of MtBGAL domain were presented. Additional file S1: All tandem and segmental duplicated *MtBGAL* gene pairs in *M. truncatula*, and the orthologous gene pairs between *M. truncatula* and *Arabidopsis* as well as *M. truncatula* and rice. Additional file S2: List of all identified cis-acting elements in all MtBGAL genes found in *M. truncatula*. Additional file S3: Detailed information on the available expression levels of *MtBGAL* genes retrieved from microarray data for *M. truncatula*, including six different tissues and stem internodes. Additional file S4: List of primers used in this study.
