# Supplementary figures and images for: Genome-Wide Analysis of Glycoside Hydrolase Family 35 Genes and Their Potential Roles in Cell Wall Development in Medicago truncatula
Source: Plants (Basel). 2021 Aug 10;10(8):1639. doi: 10.3390/plants10081639 (PMC8401519; doi:10.3390/plants10081639)

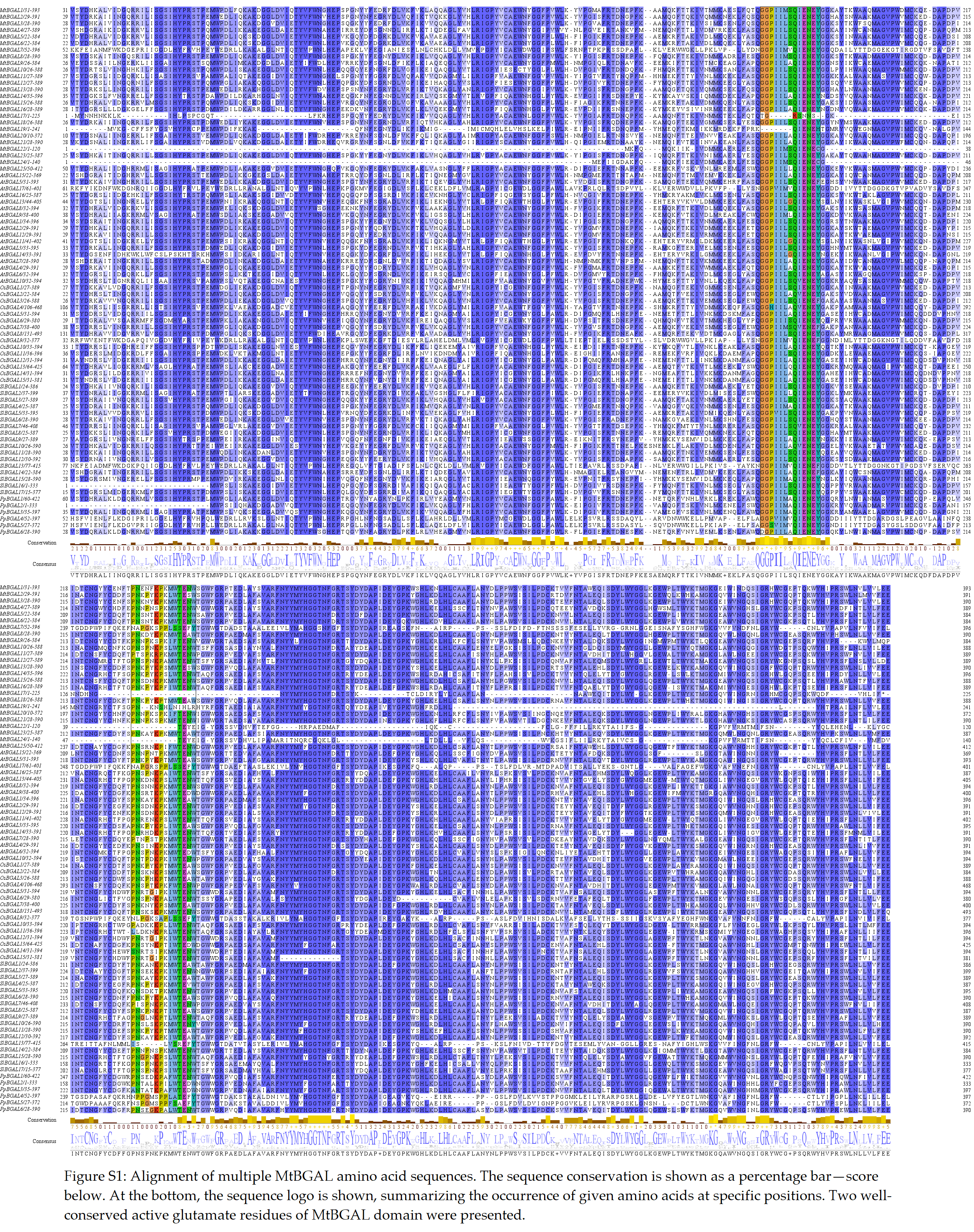

Supplement: Supplementary file 1 [file plants-10-01639-s001.zip › Figure S1.png]
